# How I do it — asleep DBS placement for Parkinson’s disease

**DOI:** 10.1007/s00701-023-05659-7

**Published:** 2023-06-15

**Authors:** Pedro Roldan, Alejandra Mosteiro, Francesc Valldeoriola, Jordi Rumià

**Affiliations:** 1 Department of Neurosurgery, Hospital Clínic de Barcelona, Barcelona, Spain; 2 Department of Surgery, Faculty of Medicine, Universitat de Barcelona, Barcelona, Spain; 3 Department of Neurology, Hospital Clínic de Barcelona, Barcelona, Spain

**Keywords:** DBS, Parkinson, Subthalamic nucleus, Asleep, OARM

## Abstract

**Background:**

Traditionally, functional neurosurgery relied in stereotactic atlases and intraoperative micro-registration in awake patients for electrode placement in Parkinson’s disease. Cumulative experience on target description, refinement of MRI, and advances in intraoperative imaging has enabled accurate preoperative planning and its implementation with the patient under general anaesthesia.

**Methods:**

Stepwise description, emphasising preoperative planning, and intraoperative imaging verification, for transition to asleep-DBS surgery.

**Conclusion:**

Direct targeting relies on MRI anatomic landmarks and accounts for interpersonal variability. Indeed, the asleep procedure precludes patient distress. A particular complication to avoid is pneumocephalus; it can lead to brain-shift and potential deviation of electrode trajectory.

**Supplementary Information:**

The online version contains supplementary material available at 10.1007/s00701-023-05659-7.

## Relevant anatomy

**Fig. 1:**
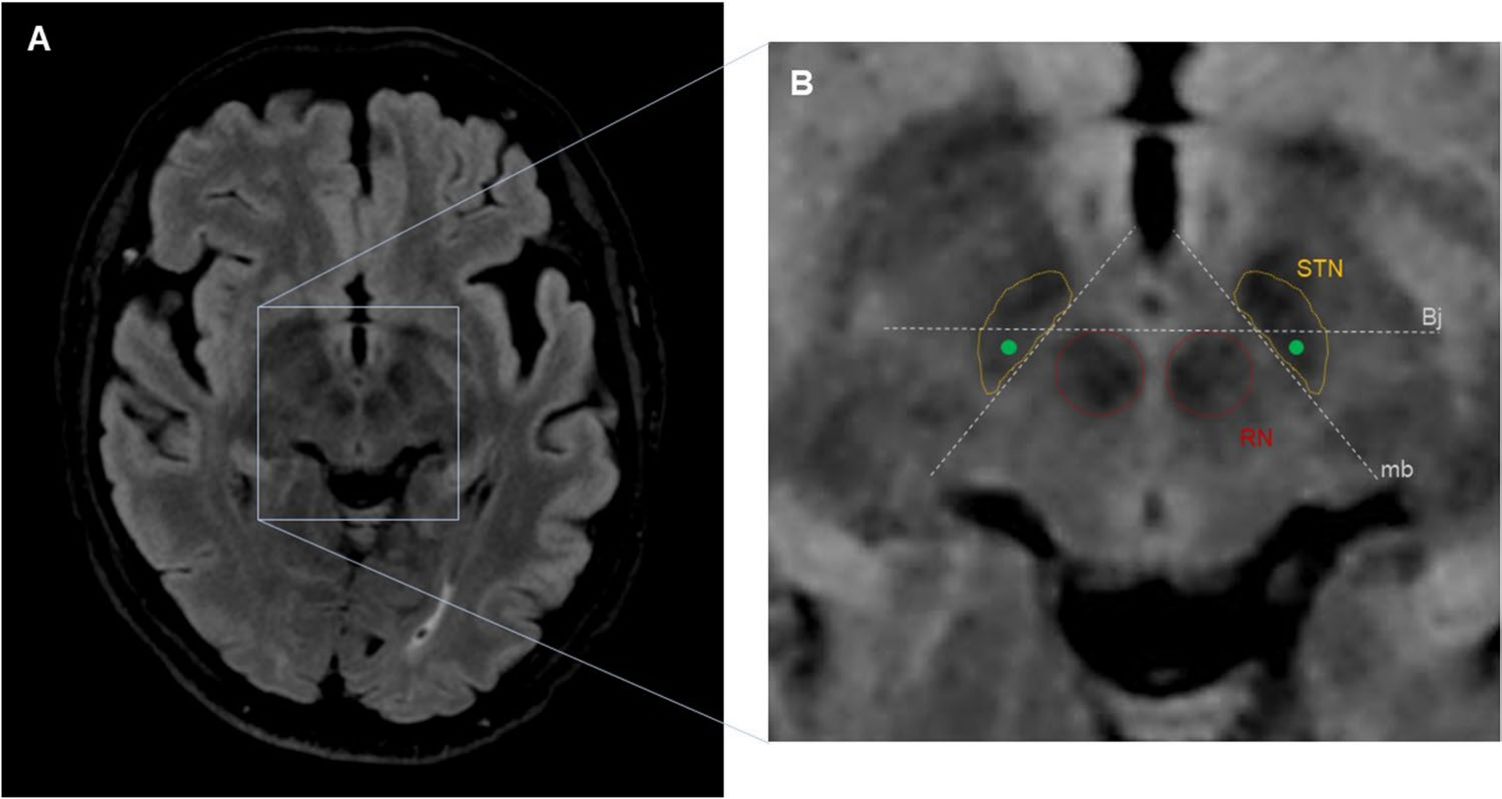
Schematic representation of the anatomic landmarks for direct targeting of the subthalamic nucleus (STN). Fluid-attenuated inversion recovery MR image, axial slice at the level of the mesencephalon. The target (green solid circle) is situated at the dorsolateral STN, approximately 1 mm posterior to the Bejjani line (Bj) passing on the anterior border of the red nucleus (RN), and 1.5 mm lateral to the medial border (mb) of the STN

The subthalamic nucleus (STN) is a lens-shape nucleus, near the substantia nigra and the red nucleus (RN) (Fig. 1). The STN motor region is posterolateral, and undesired effects of stimulation may occur if the anteromedial area or the adjacent corticospinal tract are affected [2]. Traditional indirect targeting, based on stereotactic atlases, did not account for individual variability, and thus, intraoperative verification with micro-registration of neuronal activity and clinical exploration were necessary.

Direct targeting relies on MRI anatomic landmarks and accounts for interpersonal variability. Indeed, direct DBS targeting and electrode placement under general anaesthesia is as accurate and safe as conventional awake surgery [4, 5], while precluding patient distress and being logistically more convenient [6, 7].

## Technique

### Preoperative planning

Three sequences are needed for preoperative planning — angio-CT, FLAIR, and T1-contrast-enhanced (T1 + C) — plus optional DTI for tractography reconstruction [3]. Angio-CT is selected as the reference study, to make fusion of preoperative planning with intraoperative CT more accurate. 3 T MRI and double dose of contrast agent are preferred for high resolution images of the basal ganglia and small parenchymal vessels.

In the working station, angio-CT is fused with MRI sequences. FLAIR is selected for anatomical targeting, and images are reformatted to the AC-PC plane. Conventional coordinates for STN are *X* = 11, *Y* =  − 2.5, *Z* =  − 4.5; however, we proceed with direct anatomical targeting [3]. Saturate FLAIR attenuation window so the shape of RN and STN become more evident.

The level at which the RN has its maximum diameter is selected, usually around *Z* =  − 4.5 or − 5. Note that this level could be different in each side. The Bejjani line is traced passing by the anterior border of the RN, extending towards the STN. Set your target about 1 mm posterior to Bejjani line and 1.5 mm lateral to the medial border of STN.

**Fig. 2:**
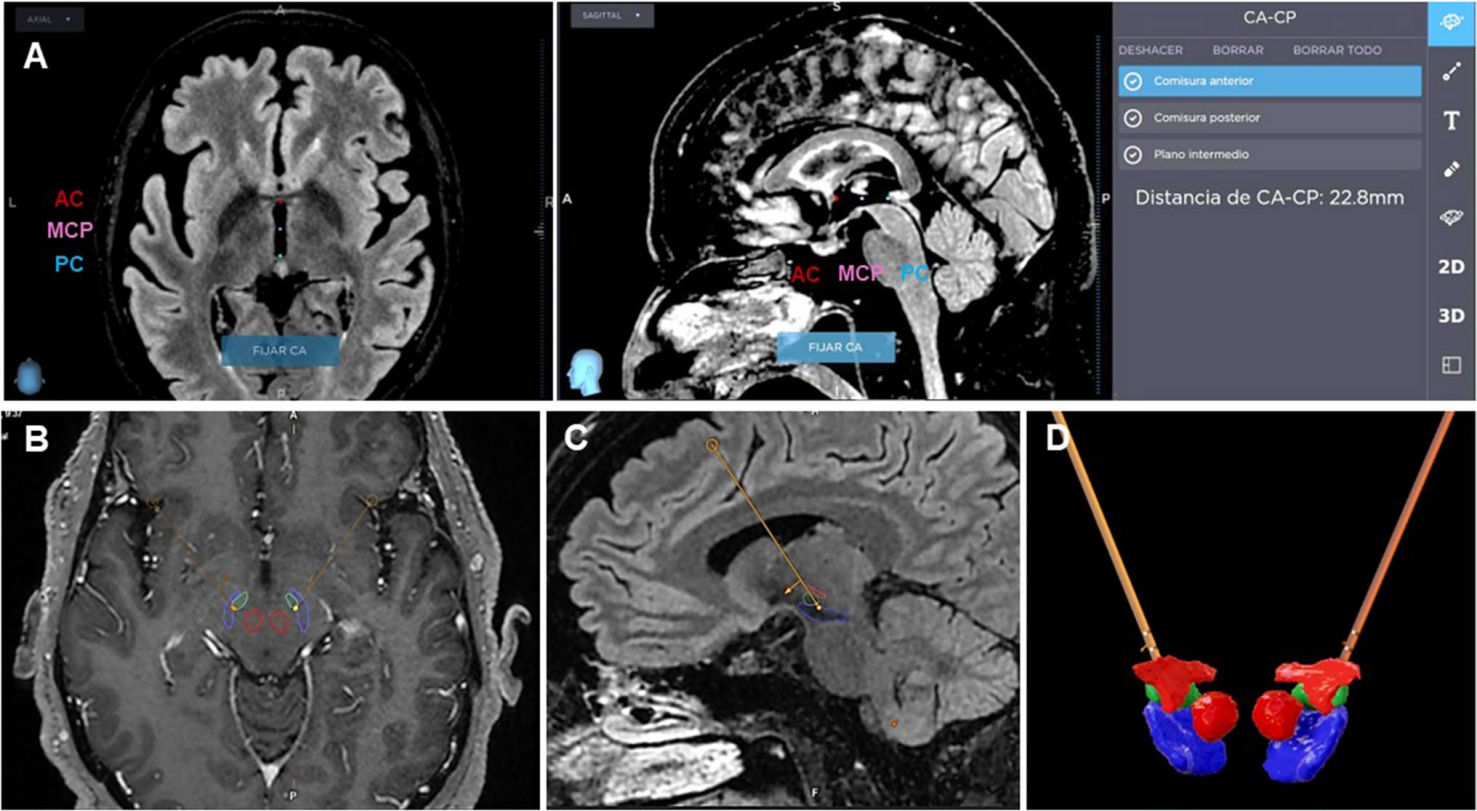
Targeting and preoperative planning. **A** Defining the anterior commissure-posterior commissure (AC-PC) line. The AC is marked at its posterior border. The PC is marked where it bulges towards the third ventricle, just above the beginning of the aqueduct. In the axial sections, the AC-PC line should pass through the interhemispheric fissure. **B** The Bejjani line is traced passing by the anterior border of the red nucleus, at the level were this nucleus shows its maximal diameter. If an anterior protrusion is identified, this is dismissed for tracing the aforementioned line. **C** The target is set 1 mm posterior to the Bejjani line and 1.5 mm laterally to the medial border of the STN. **D** The entry point is usually located 10 mm anterior to the coronal suture. **E** The arch is selected so that the trajectory traverses the STN from top to bottom. **F** The angulation is set so that the entry is through a gyrus and avoids *en passant* vessels. **G** A 3D reconstruction of the electrodes, traversing the STN in its whole longitude, and avoiding the internal capsule laterally

Now shift to T1 + C to select the entry point, usually 10-mm anterior to the coronal suture. Select the arch so the trajectory traverses the STN from top to bottom, and the angulation so the entry is through a gyrus and skirts *en passant* vessels. Avoid extreme arches that would transect the temporalis muscle. Verify the safety of the complete trajectory. 3D reconstruction of the electrodes within the STN helps confirm the accuracy of planning (Fig. 2).

### Intraoperative procedure: electrode placement

**Fig. 3:**
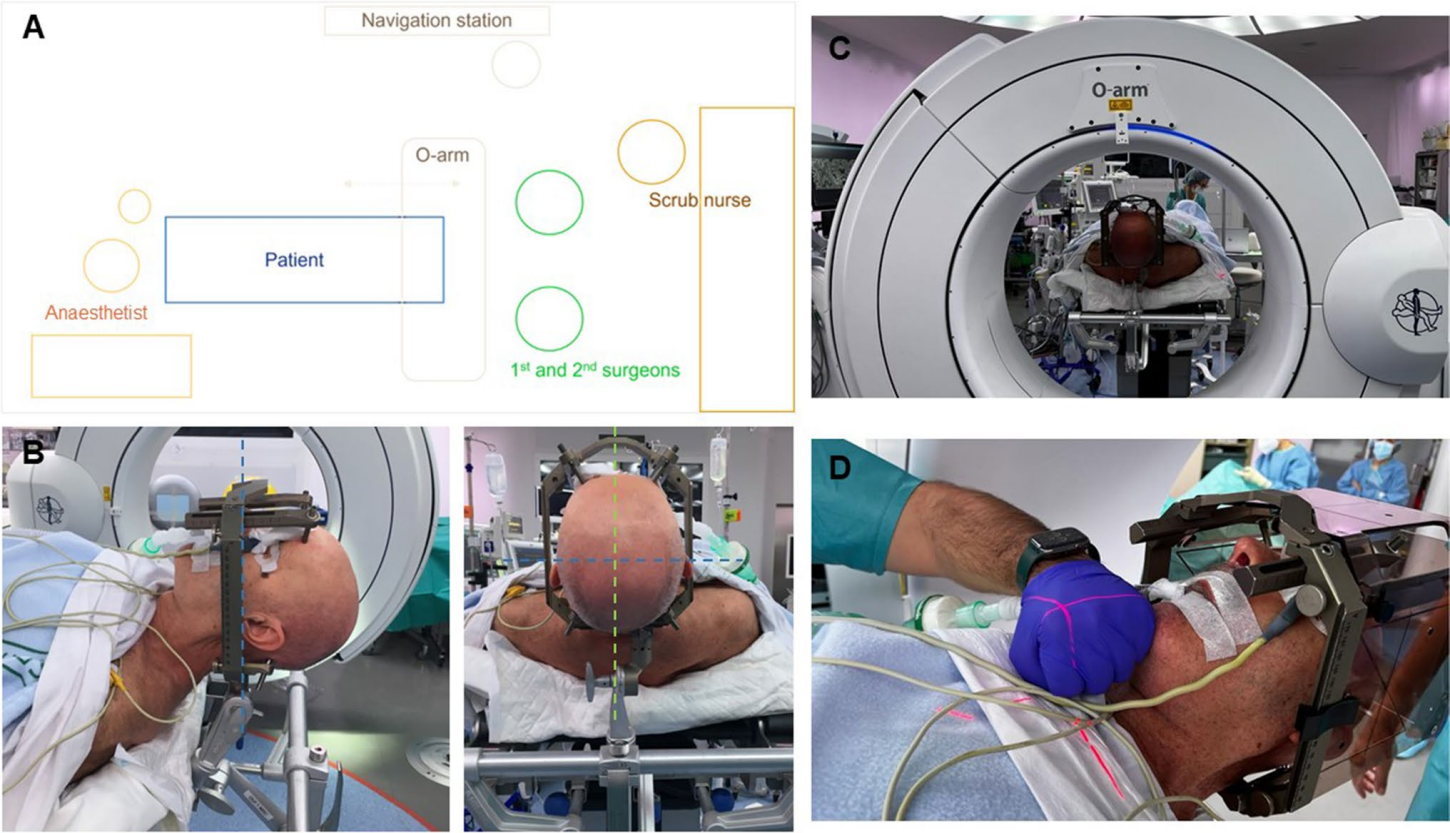
Room setting and patient positioning. **A** Standard operating room set up. **B** Stereotactic frame placement, centred on the midline (green dashed line) and parallel to the intermeatal line (blue dashed line). **C** The head-holder fixation arm should be as packed as possible, to facilitate O-arm movements. **D** The O-arm position is adjusted, so that the laser lights coincide with the midline, the external acoustic meatus and about one fist below the chin to include the whole skull base. The parameter for CT acquisition are set (supine position, 3D, stereotactic, 120 kV, 20 mA, 40 cm field, 150mAS)*,* and the examination and parking positions are established. After the first CT is acquired, the O-arm is moved to the parking position, and the surgical site is draped and prepared

Standard room set up is shown in Fig. 3. Patient is supine, with the head slightly elevated. Stereotactic frame is centred, with the nasion and the external auditory meatus as main references. O-Arm®2 fluoroscopic device (Medtronic Inc., Minneapolis, MN, USA) is brought to exploration position; both skull base and calvarium are included. Scalp is disinfected twice, first with iodine soap and then with iodine solution. Since 2014, we avoid hair shave for better cosmetic result and have not seen an increase in surgical site infections. The frame is disinfected with alcoholic solution, and the surgical site draped.

**Fig. 4:**
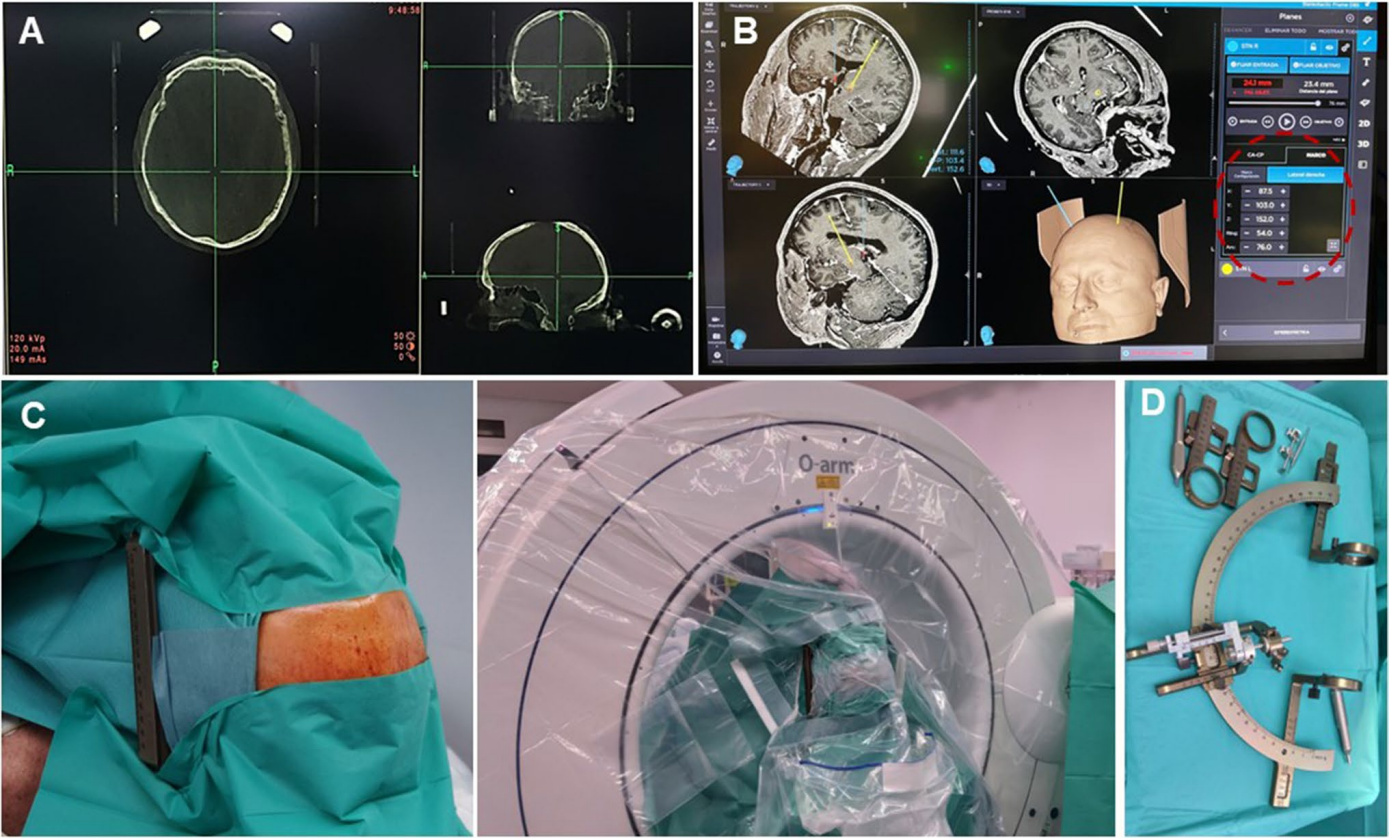
Intraoperative programming and preparation. **A** The intraoperative CT 3D image is fused with preoperative planning, and **B** coordinates are changed from AC-PC reference to frame-based reference. Adjustments are needed to entire figures or 0.5 decimals. **C** The field is draped, leaving the laterals of the frame uncovered. **D** The coordinates are transferred to the stereotactic frame

**Fig. 5:**
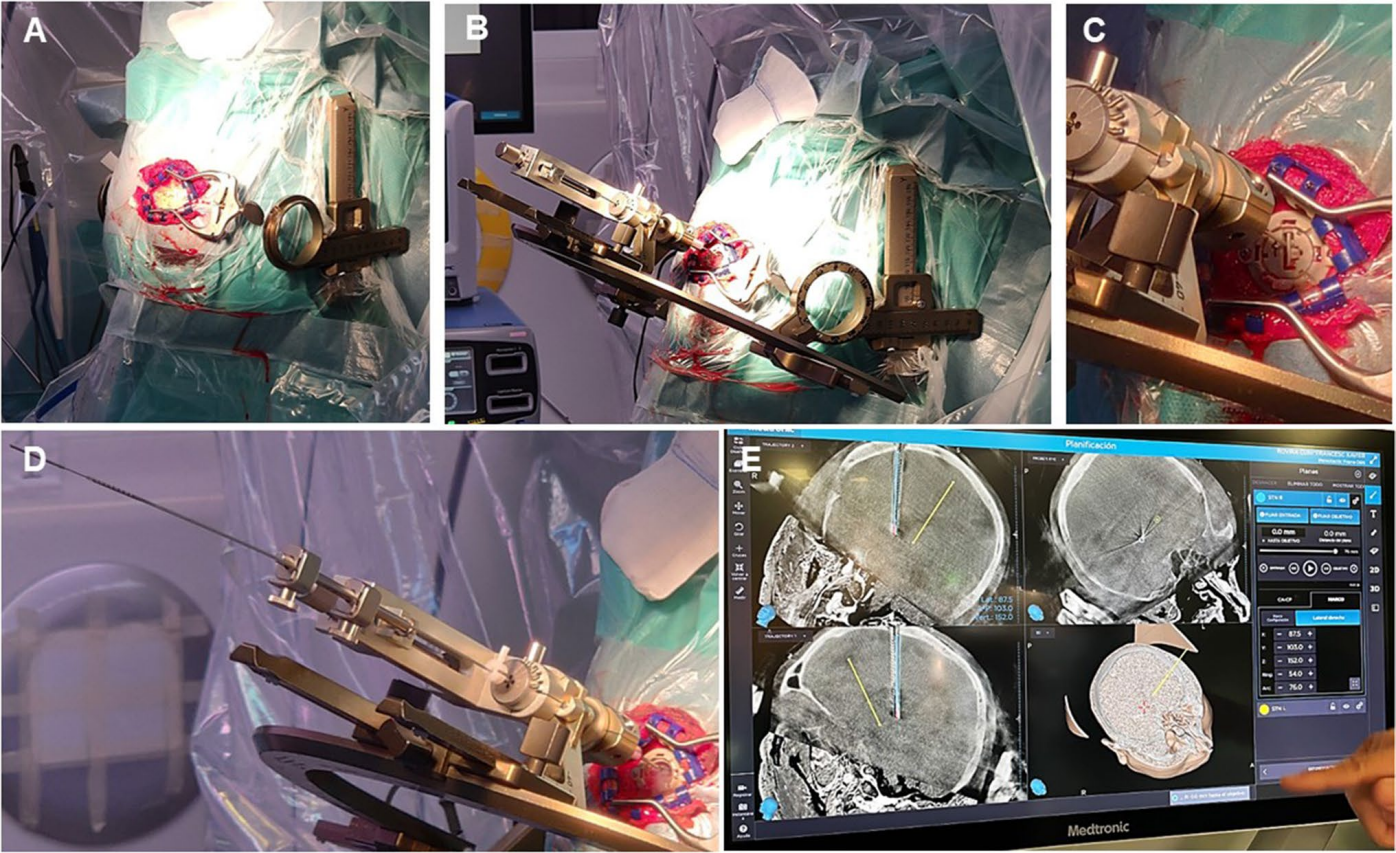
Electrode placement. **A** 3-cm linear incision is made over the marked point of entry. **B** The point of the trepanation is indicated with the aid of the frame. **C** The fixation system is anchored to the burr hole and the dura is opened in a cruciate fashion. Cautious opening of the arachnoid and cerebral cortex will avoid catheter deviation during the introduction. Once the canula is in place, a tissue sealer is applied on the burr hole to prevent CSF outflow and pneumocephalus. **D** The directional electrode is placed within the canula, with the appropriate orientation, and the canula is removed. F) A second CT is obtained and fused with the preoperative planification to check for the accuracy of the target. If satisfactory, the lock is laid, and the distal portion of the catheter, protected and tunnelled towards the parietal eminence

A basal 3D-intraoperative stereotactic O-Arm®2 sequence is acquired and fused with preoperative images. Pre-planned coordinates (referenced to AC-PC line) are now referenced to the frame and need to be adjusted to 0.5 figures. Cautious verification of the target and whole trajectory is paramount at this step. Selected coordinates are set in the frame (Fig. 4). Entry point is marked in the skin, and 3-cm linear incision is made (Fig. 5). Dissect the subcutaneous plane towards the right parietal eminence and contralateral entry point will faccilitate for later cable tunnelling. The point of trepanation is guided by the frame. Fixation system is anchored to the burr hole, dura is incised in a cruciate fashion, and arachnoid and cortex are carefully cauterised. Canula is inserted directly to target point, avoiding traction of the arachnoid at the surface as this could deviate the trajectory. Tissue sealer is applied on the burr hole to prevent CSF outflow and pneumocephalus. The electrode is inserted within the canula. If using directional electrodes, beware of their orientation. Monopolar cautery is restricted from this point on.

The canula is carefully removed and intraoperative 3D-stereotactic O-Arm sequence is acquired. This second image is fused with preoperative planification to check the accuracy of the target; we would allow 0.5 mm deviation from the desired position; otherwise, the electrode is withdrawn and replaced. If the position is satisfactory, the lock is laid, and the distal portion of the catheter protected and tunnelled towards the parietal eminence. This is a critical step, as unnoticed pull-out could occur. After the first electrode has been placed, the 3D-stereotactic O-arm sequence serves to rule out complications or significant brain shift in the contralateral hemisphere that would jeopardize the safe and accurate implantation of the contralateral electrode. If none of these are detected, the same process of implantation is repeated in the contralateral side. Wounds are sutured.

### Intraoperative procedure: generator placement

**Fig. 6:**
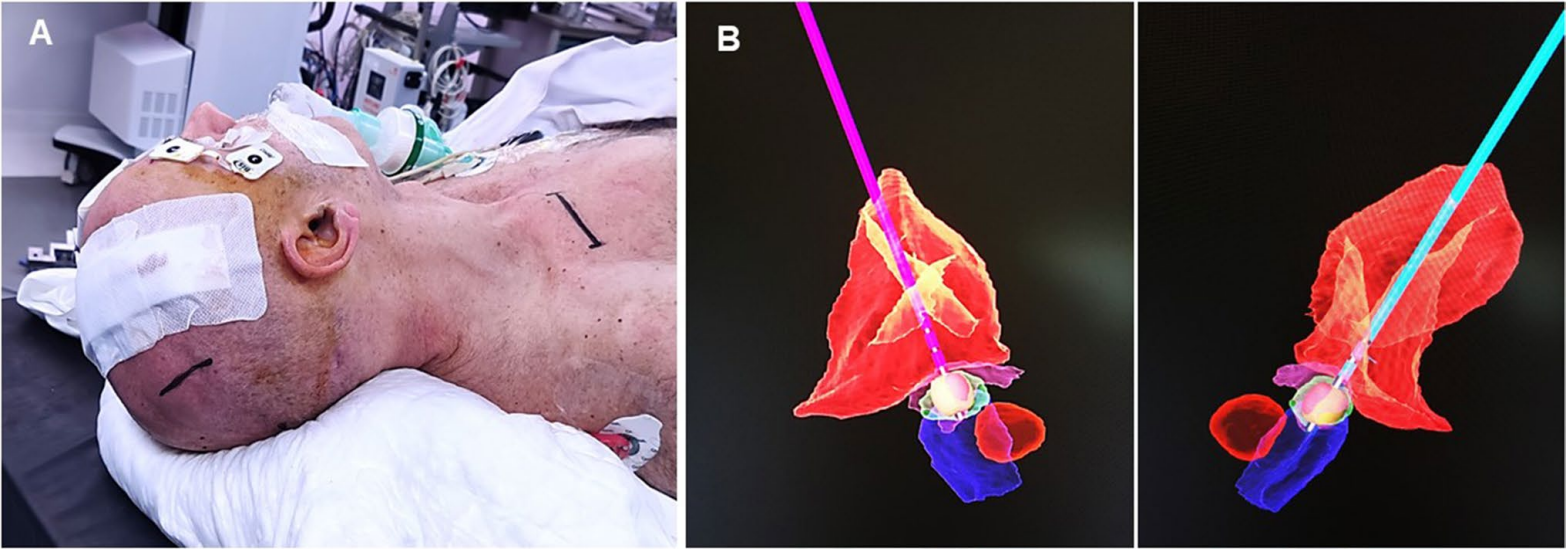
Placing the generator. **A** The right shoulder is elevated and the head rotated 90° towards the left. The incision is marked 1 cm below the right clavicle. A subcutaneous pocked is opened in the infraclavicular region, over the pectoralis major fascia. Then, an incision in the parietal eminence reveals the distal end of the electrodes. The extension cables are tunnelled between the two incisions and connected to the electrodes and to the generator. After placing the generator in the subcutaneous pocked impedances are obtained. **B** A 6-h postoperative scan is obtained to rule out complications and to check the final position of the electrodes. A 3D reconstruction can be obtained showing the relation between the electrodes, the area of stimulation, and the subthalamic nucleus

O-arm and frame are removed; the patient is placed with the right shoulder elevated and the head rotated 90° contralaterally. The incision to allocate the generator is marked 1 cm below the clavicle, on the right side (left side should be kept intact in case a cardiac pacemaker is needed in the future) (Fig. 5). The field is again prepared and draped. A subcutaneous pocked is created over the pectoralis major fascia. Then, an incision in the parietal eminence reveals the distal end of the electrodes. Extension cables are tunnelled between both incisions and connected, taking care to place the right and left electrodes in the appropriate channel. After placing the generator in the subcutaneous pocket, impedances are checked. High impedance values may indicate incorrect connection between components, presence of fluid within the cables, cable damage, or hematoma in the targeting site. If impedances are correct, wounds are closed. If impedances are high, the surgeon should actively seek for any misalignment and/or interfering debris (i.e., blood) between lead and extension contacts, and at the connection between the extension and the neurostimulator itself. The patient is transferred to postoperative surveillance for 6 h; a control 3D-CT scan is obtained to rule out postoperative haemorrhage and verify the final position of the electrodes (Fig. 6).

## Indications

Indications for asleep-DBS for Parkinson’s disease can be superposed to the standard awake intervention.

## Limitations

The asleep procedure can be extended to other anatomical targets provided reliable identification on MRI, such as internal globus pallidus (Parkinson’s, dystonia), anterior thalamic nucleus (epilepsy), and newer psychiatric indications. However, there are still limitations to MRI resolution requiring an awake intervention, like the thalamic ventral intermediate nucleus in essential tremor.

Confirmation of adequate targeting and spearing of nearby structures can only be confirmed after surgery. Therefore, suboptimal outcomes or undesirable effects would require a revision surgery. Advanced neuroimaging modalities like DTI may assist in lead placement, optimising direct anatomical targeting.

## How to avoid complications

Major complications are intraparenchymal haematoma and infection. Other distinctive problems can be a concern, mainly pneumocephalus, bow-neck syndrome, and adverse effects of stimulation.

Preoperative planning is paramount to avoid intraoperative bleeding. Trajectory should start at a gyrus (not a sulcus) and avoid trespassing the ventricles. Trajectory should be planned with T1 + C, with a security margin of ≥ 1 mm from any vessel. Use of “probe’s eye” vision helps verifying the chosen trajectory.

Pneumocephalus should be avoided, as a major contributor to brain-shift potentially provoking trajectory deviation [8]. To this aim, the head should only be slightly elevated; corticotomy and time to canula introduction should be limited, and tissue sealer should be applied once the canula is in place.

Bowstringing from extension cables causes pain and tension in the neck. Therefore, head should be rotated 90° contralaterally, and tunnelling should aim towards the sternoclavicular joint, instead of directly towards the middle-clavicular point [1].

Finally, preventing adverse effects of the stimulation relies in three fundaments: 1) Precise preoperative targeting. 2) Confirming the target after intraoperative correction of stereotactic coordinates referenced to the frame. 3) “Getting to know your frame”: most stereotactic frames, as any other measuring device, have an inherent systematic error. Identifying this systematic deviation from the preselected coordinates can play in your favour when adjusting the coordinates to achieve the desired positioning. The use of directional electrodes provides the versatility of shaping the electric field. Compared to conventional cylindrical electrodes, directional ones have a radial segmentation of the contacts permitting to divert the electric field in a particular direction. This overcomes certain deviation from the optimal target position, thus improving the therapeutic window and reducing adverse effects.

## Specific perioperative considerations

We normally allow a week before turning on the stimulation and beginning programming. During that time-lapse, we aim to reduce the antiParkinsonian medication to facilitate identification of the most favourable effects with the stimulation parameters.

## Specific information to give to the patient

At discharge, patients are given a detailed list of precautions now that they bear a neurostimulator (Supplementary material).

## Supplementary Information

Below is the link to the electronic supplementary material.

Supplementary file1 (DOCX 14 kb)

## Abbreviations

AC: Anterior commissure
CT: Computed tomography
DBS: Deep brain stimulation
FLAIR: Fluid-attenuated inversion recovery
MRI: Magnetic resonance imaging
PC: Posterior commissure
STN: Subthalamic nucleus
