# Supplementary material for: How I do it — asleep DBS placement for Parkinson’s disease
Source: Acta Neurochir (Wien). 2023 Jun 15;165(8):2189–95. doi: 10.1007/s00701-023-05659-7 (PMC10409652; doi:10.1007/s00701-023-05659-7)
Supplement: Supplementary file 1 — Supplementary file1 (DOCX 14 kb) [file 701_2023_5659_MOESM1_ESM.docx]

**Supplementary material. How I do it - Asleep DBS placement for Parkinson’s disease**

Specific precautions in patients bearing a neurostimulator:

- Potential interaction with intracardiac devices. It is strongly recommended that the neurostimulator and the cardiac peacemaker or defibrillator are implanted in opposite sides of the body, to diminish the risk of the interaction between the two. Of special concern is the fact that the cardiac device may detect the neurostimulator activity, therefore the latter should be programmed in bipolar mode and with a minimum frequency of 60 Hz.
- Diathermia is contraindicated, neither short-wave diathermia nor microwave or ultrasound therapy.
- Electrocautery. If a surgery of any nature requiring electrocautery coagulation is required, the following recommendations must be noted: Deactivate the neurostimulator before the procedure. Only use bipolar cautery. If monopolar cauterisation were indispensable, low voltage must be chosen, and the trajectory of the current (i.e., the grounding plaque) must be located as far as possible from the neurostimulator.
- High frequency ultrasound and lithotripsy are discouraged. If needed, the beam should be focus with a distance superior to 15 cm from the neurostimulator.
- Radiofrequency ablation may not be safe. The electric currents may provoke and overheating of the neurostimulator and particularly at the level of the intraparenchymal electrodes.
- Antitheft detectors and other security devices. The patient should hold a specific identification card to avoid passing through security arched, a manual detection is preferable. If unavoidable, the neurostimulator should be previously turned off, and the time under the arch should be minimised.
- Laser. The interaction between laser and the neurostimulator is unlikely. Still, the laser beam should be kept as far as possible from the stimulator.
- Radiotherapy. The interaction between radiotherapy and the neurostimulator is unlikely. Nonetheless, high radiation beams should be avoided, such as cobalt 60 or gamma. If needed, a lead screen protection can be interposed between the radiation beam and the neurostimulator.
- TENS. Transcutaneous electric neurostimulation can be applied, however, poles should be placed so as that the current does not pass though the area of the neurostimulator.
